# Vitamin D status in Moroccan pregnant women and newborns: reports of 102 cases

**DOI:** 10.11604/pamj.2016.24.170.4782

**Published:** 2016-06-29

**Authors:** Fouzia Mnebhi Loudyi, Jalal Kassouati, Meryem Kabiri, Naima Chahid, Aicha Kharbach, Hassan Aguenaou, Amina Barkat

**Affiliations:** 1 Research unit for mother & Child Health and Nutrition, Faculty of Medicine and Pharmacy, University of Mohammed V, Rabat, Morocco; 2 National Referral Center of Neonatology and Nutrition, Ibn Sina University Hospital, Rabat, Morocco; 3 Laboratory of Biostatistics and Epidemiological Research, Rabat, Morocco; 4 Maternity of Rabat, Rabat, Morocco; 5 Unité Mixte de Recherche en Nutrition et Alimentation URAC 39, Université Ibn Tofail-CNESTEN, RDC-Nutrition AFRA/AIEA, Morocco

**Keywords:** Vitamin D, pregnant woman, newborn

## Abstract

**Introduction:**

Vitamin D insufficiency to pregnant women has been associated with a number of adverse consequences, and has been recognized as a public health concern. The aim of this study was to evaluate vitamin D status of Morrocan pregnant women and their newborns. Our study is being the first of its kind in Morocco, as it supports the program of systematic supplementation of pregnant women in the third quarter. Its results have established a new program for the fight against the deficit of various nutrients, thereby intake of vitamin D has become routine. So this work is a true example of action research.

**Methods:**

It’s an observational and a cross sectional study. The data was collected prospectively from the 1st January to 31 December 2012 in the labor room of the Souissi maternity hospital, at the Ibn Sina university center of Rabat in Morocco. Women included were consented to participate in the study. Data on epidemiological, sociodemographic and clinical characteristics was recolted by interview, physical exam and biochemistry parameters. Hypovitaminosis D is defined as serum level of vitamin D ≤ 50 nmol/l (20 ng/ml).

**Results:**

Our study included 102 cases of mother-newborn pairs. The average age of mothers was 28.3 +/- 6.7 years (range 17-43 years), 90.1% of women enrolled had a hypovitaminosis D, the average weight of newborns was 3377.9 +/- 509g (2270 - 4880g). Hypovitaminosis D is not correlated with the origin, season, body mass index, birth interval and birth weight. It was positively correlated with maternal serum calcium (p=0.000).

**Conclusion:**

The maternal hypovitaminosis D is real public health problem. The prevention is necessary, by the systematic vitamin D supplementation for pregnant women.

## Introduction

Vitamin D or 1,25 dihydroxyvitamin D or calcitriol is an essential fat soluble vitamin that comes from two sources: exogenous (diet and supplements) and endogenous (skin photosynthesis) [1]. Its regulation is made by acting hormones, direct or indirect way [2], in addition to autoregulation mechanism depending on the calcium and phosphorus blood levels. Vitamin D has many essential roles: metabolism of calcium and phosphorus, mineralization of bones, reduction the risk of cancers (breast, prostate, colon … etc) [3], prevention of autoimmune diseases, metabolic and neurologic disorders [4–6]. Hypovitaminosis D is defined as serum level of vitamin D ≤ 50 nmol/l (20 ng/ml) [7]. During pregnancy, changes are occurring in maternal vitamin D and calcium metabolism.

It allows to the fetus to establish his own pool of vitamin D and calcium. Almost 30 g of calcium is accumulated; 80% of this quantity is provided in the last trimester of pregnancy. The serum concentration of vitamin D increases from early pregnancy; to reach its peak in the third trimester. The serum concentration of calcium is elevated in foetus more than mother. Unlikely, vitamin D is more elevated in the maternal blood. The regulation of vitamin D and calcium during pregnancy is influenced by many factors: PTHrp (PTH related peptide) produced by fetal parathyroid gland and placenta, prolactin, placental lactogenic hormone, calcitonin, osteoprotegerin and estrogen [8]. The aim of this study is to evaluate vitamin D status of Morrocan pregnant women and their newborns. Our study is the first of its kind in Morocco, as it supports the program of supplementation of pregnant women in the third quarter. Its results have established a new program for the fight against the deficit of various nutrients. Thereby, intake of vitamin D has become systematic.

## Methods

### Type of study

It’s an observational and a cross sectional study. The data was collected prospectively from the 1^st^ January to 31 December 2012 in the labor room of the Souissi maternity hospital, at the Ibn Sina university center of Rabat in Morocco.

### Study population

The recruitment was conducted over 12 months, by four samples according to four seasons. 102 cases of mother-newborn pairs were collected. Sample selection was randomized: we collect the data every Thursday and Sunday of each week. Sample size was determined by the number of available biochemical reagents.

### Inclusion criteria

Healthy women without chronic disease or pathologic pregnancy, admitted for full term singleton pregnancy, willing, in labor room of Souissi maternity in Rabat.

### Data collection

Epidemiological, sociodemographic, clinical characteristics and biochemistry parameters were collected. Serum concentration of different parameters was measured for each participant pregnant lady by venous blood sampling at admission moment in the labor room, and for her newborn by umbilical cord blood sampling after birth. The samples were identified, centrifuged and frozen in less than 20 degrees, and sent the next day to the laboratory. We studied these parameters: calcium, phosphorus, alkaline phosphatase and vitamin D.

### Measurement of 25-(OH) vitamin D

The measurement of serum 25 OH vitamin D is made by chemiluminescence. During the first incubation, 25 OH Vitamin D is dissociated from its binding protein and binds to the specific antibody on the solid phase. After 10 minutes the tracer, (vitamin D linked to an isoluminol derivative) is added. After second 10 minutes incubation, the unbound material is removed with a wash cycle. Subsequently; the starter reagents are added to initiate a flash chemiluminescent reaction. The light signal is measured by a photomultiplier as relative light units (RLU) and is inversely proportional to the concentration of 25 OH Vitamin D present in calibrators, controls, or samples. Hypovitaminosis D is defined as serum level of vitamin D ≤ 50 nmol/l (20 ng/ml).

### Data entry and analysis

After being collected in tables, the data transferred into Access (and then into) Excel system. The data was processing and analyzed by use of SPSS 13.0 software. Quantitative variables expressed in mean, standard deviation and median. Qualitative variables expressed in frequency and percentage. The significance threshold fixed at p < 0.05.

### Ethics

The women were recruited in the labor room and they were provided with written information and signed an informed consent. The Ethics Committee of the faculty of medicine and pharmacy of Rabat approved the study.

## Results

Our sample contained of 102 mother - newborn pairs (i.e. 204 samples) distributed over four groups (four different seasons): spring, summer, autumn and winter.

### Maternal data

**Figure 1:**
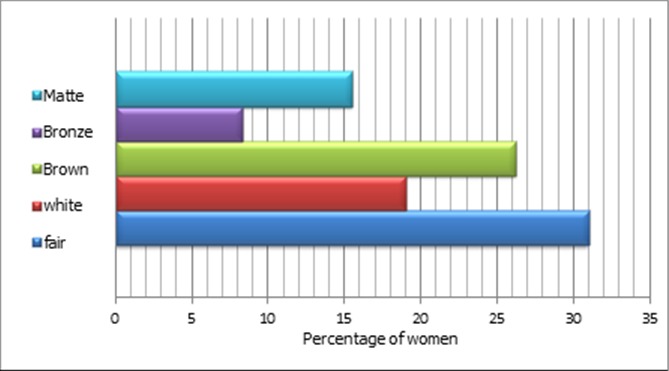
Distribution of patients according to the skin color

The average age of mothers was 28.3 +/- 6.7 years, with minimum age of 17 years and maximum of 43 years. Of 102 mothers, 85 were from urban and 17 from rural areas. The average age at marriage was 22.4+/- 5 years and at first pregnancy was 23.26 +/- 5 years. The average gravidity number found 2.08 +/- 1.3 and that of parity was 1.88 +/- 1.16. 45 patients or 48.1% of sample population had used contraceptive methods, among them 44.1% with oral method. The median duration of contraceptive was 12 months. More than two thirds of the women wore the veil, and 31% of them had fair skin, while 26.2% were brown skinned (Figure 1). The average of daily exposure to sun is estimated to 1.54 hours +/- 1 with minimum of 0 hour and maximum of 6 hours. It seems to depend on dressing way up.

**Figure 2:**
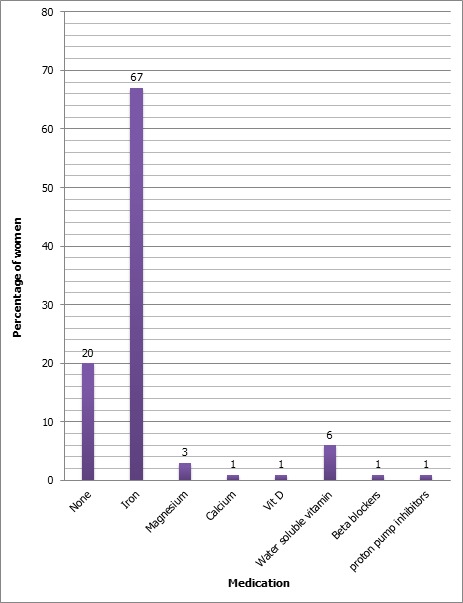
Taking medication during pregnancy

75 women had taken some medicines during the pregnancy, with median duration of 3 months, the minimum was 1 month and maximum was a 9 months. One woman had taken the vitamin D in the 3rd trimester with dose of 200 000 IU as single dose (Figure 2).

**Figure 3:**
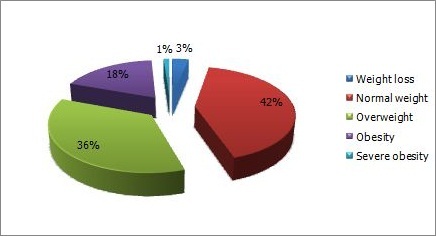
BMI before pregnancy

The average weight of the women before the conception was 65.91 +/- 11.91 kg. At the moment of childbirth it was 73.35 +/- 13.21 kg (Table 1). Analysis of pre-pregnancy BMI shows that 41.9% of women had a normal weight (Figure 3).

**Table 1:** Anthropometric measurements of patients

	Number	Minimum	Maximum	Average	Standard deviation
Height (m)	100			1.60	0.056
Weight before pregnancy (kg)	93	45	95	65.91	11.91
Weight after pregnancy (kg)	100	48	118	73.35	13.21
Weight gain during pregnancy (kg)	93	-16	29	7.97	5.952
Waist circumference (WC) (cm)	46	70	118	91.28	14.281
Hip circumference (HC) (cm)	46	82	126	102.00	9.773
W/H ratio	43	1	1	0.85	0.097
Mid upper arm circumference (cm)	46	22	36	29.34	4.23

### Neonatal data

The mode of delivery of newborns was vaginal delivery in 72% of cases and cesarean section in 28% of cases. 53 of newborns were male and 47 were female. The average birth weight was 3377.9+/-509g, average height 49.18 +/- 3.3 cm and average head circumference 34.75 +/- 2.2 cm.

### Phosphocalcic status

The average serum concentration of vitamin D was 11.06 ng / ml for mothers, and 10.13 ng / ml for newborns (Table 2). Hypovitaminosis D was noticed in 90.1% of women and 92.9% of newborns. Hypocalcemia was detected in 39.6% of mothers and nearly in 4% of newborns. Over 95% of newborns had hyperphosphatemia, while an elevated alkaline phosphatase (ALP) was recorded in 78.22% of the mothers and in 80% of newborns.

**Table 2:** Phosphocalcic status of mother and newborn (Vit D, Ca, P, ALP)

	Number	Minimum	Maximum	Average	Standard deviation
Mother calcium (Ca M) mg/l	101	65	120	85.84	10.38
Mother phosphorus (P_M) mg/l	101	20	203	53.43	33.89
Mother ALP (IU)	101	56	720	205.96	87.79
Mother vitamin D (Vit D_M) µg/l	101	4	32	11.06	5.91
Baby serum calcium (Ca B)	70	52	144	99.70	12.55
Baby serum phosphorus (P_B)	70	42	304	112.35	61.26
Baby ALP	70	2	531	186.54	81.35
Baby serum vitamin D (Vit D_B)	70	3	42	10.13	6.78

### The correlation between vitamin D status and maternal and neonatal data

The study findings revealed that maternal vitamin D deficiency is not correlated with the origin, season, body mass index, and birth interval and birth weight. It is significantly associated with maternal serum calcium (p=0,000) (Table 3).

**Table 3:** Correlation between maternal vitamin D status and different variables

	n	p
**Origin**	102	0,598
Urban	85	
Rural	17	
**Dressing**	90	0,361
Not veiled	19	
Veiled	71	
**Skin color**	84	0,345
Fair skin	26	
White	16	
Brown	22	
Bronze	7	
Matte	13	
**Daily exposure to the sun**	82	0,130
**Season**	102	0,087
Spring	22	
Summer	32	
Autumn	23	
Winter	25	
**BMI**	92	0,836
Wasting	3	
Normal weight	38	
Overweight	33	
Obesity	17	
Severe obesity	1	
**Age**	101	0,312
**Birth interval**	51	0,314
**Maternal serum calcium**	101	0,000
**Maternal serum phosphorus**	101	0,482
**Maternal alkaline phosphatases**	101	0,872

## Discussion

This study showed that 90.1% of pregnant women had hypovitaminosis D with average rate of 11.09+/- 5.91 ug / l, which is significant rate of vitamin D deficiency. The finding of this study is close to that of SHIBATA et al. [9] with 89.5% of hypovitaminosis and of TAO et al [10] which showed the deficiency in vitamin D in 90.5% of study population ( 1695 pregnant women ). Vitamin D status of babies depends on the reserve established almost in first 6 to 8 weeks of intrauterine life [8]. This explains the significance of hypovitaminosis D observed in the newborns. These results indicate the importance and the emergency need for vitamin D supplementation for all pregnant women in this country.

Our sample distributed over four seasons: spring, summer, autumn and winter. No difference in vitamin D status in different seasons. The study population consisted of young mothers aged from 17 to 43 years, with an average age of 28.3 +/- 6.7, which is comparable with that of various studies. The average age found 28.12 +/- 4.18 years in study of TAO et al [10]. In study of SHIBATA et al [9], the population aged between 18 and 45 years with an average age of 31.4 +/- 5.5. PERAMPALAM et al [11] reported that the average age was 30.8 +/- 5.6 years at Canberra and 27.9 +/- 6.6 years at Cambell town.

The phenomenon affected all age groups in our study population (p = 0.312), irrespective of age. Similar results were found in all cited studies [9–11]. Hypovitaminosis D is independent of birth interval (p = 0.314) and multiparity (p = 0.579). The result is similar to that of TAO et al. [10]. The body mass index (BMI) was inversely proportional to vitamin D status; the higher is the BMI, the higher is the risk for calcidiol deficiency [10–12]. Our sample included 41 overweight women and 37 obese women. The hypovitaminosis D rate was 83.33% and 85.29% in overweight and in obese women respectively. However the BMI is not retained as risk factor in this study, perhaps due to high prevalence of vitamin D deficiency that affected the majority of the pregnant women involved. The average of daily sun exposure among the participant women was 1.54 +/- 1 hours, despite it is quite enough time, more than 9 of 10 women suffered from the vitamin D insufficiency, perhaps due to low quality of exposure. The duration needed for effective sun exposure is longer than 30 minutes with uncovered arms up to shoulders. These criteria are rarely met in our study population, as more than 2/3 of participant women wore the veil. Covering dress habits is recognized predisposing factor for hypovitaminosis D [13–15]. Similar results were found in Australia [11] where more than 66.5% of population study had exposed to the sun more than 3 hours / day and hypovitaminosis was independent to sun exposure. In another study in Martinique [16] in last summer, a significant impact of sun exposure on vitamin D status was reported; 2/3 of the patients wasn’t exposed to the sun in last summer and they had low level of 25 OH D, so this proportion was reduced to the 1/3 in case of exposure (p < 0.05). In this study, the duration of sun exposure is not correlated to vitamin D deficiency. The skin pigmentation was found not significantly related with hypovitaminosis D in our study population (p=0.345), which could be explained by the frequency of the vitamin D insufficiency in our population. Bodnar et al [17] had compared 200 white women and 200 African - American women. This study showed a predominance of hypovitaminosis D in dark skinned people.

No correlation found between hypovitaminosis D and birth weight of the newborns involved in this study (p = 0.313). Nevertheless, the study of Kalra et al [18] showed a proportional relationship between the values of vitamin D and the anthropometric measurements of newborns (birth weight, birth length and head circumference).

## Conclusion

The maternal vitamin D deprivation is a real public health problem. The impact of deficiency is found in both of mother and newborn health status. Our study is the first of its kind. Its Results have established a new program to fight against the deficit of different micronutrients. The supplementation by vitamin D has become systematic for pregnant women at the third trimester in Morocco. This study is a true example of research-action in our country.

### What is known about this topic

- The vitamin D deficiency is a public health problem. When it occurs during the 1000 days of opportunity is a major challenge for health. Knowledge of the state deficit in a given population can implement effective actions to address it.

### What this study adds

- This study highlight the knowledge of the state deficit in mothers and newborns in Moroccan population for the first time in morocco;
- And also analysis of risk factors is action research for the development of prevention.

## References

[1] Holick MF (2007). Vitamin D deficiency. N Engl J Med.

[2] Holick MF, Carabedian M, Favus MI (2006). Vitamin D: photobiology, metabolism, mechanism of action, and clinical applications. Primer on the metabolic bone diseases and disorders of Mineral Research.

[3] Maalmi H, Ordonez-Mena JM, Schottker B, Brenner H (2014). Serum 25-hydroxyvitamin D levels and survival in colorectal and breast cancer patients: Systematic review and meta-analysis of prospective cohort studies. Eur J Cancer.

[4] Mitri J, Pittas AG (2014). Vitamin D and Diabetes. Endocrinol Metab Clin North Am.

[5] Dhesi JK, Jackson SH, Bearne LM, Moniz C, Hurley MV, Swift CG (2004). Vitamin D supplementation improves neuromuscular function in older people who fall. Age Ageing.

[6] Souberbielle JC, Prie D, Courbebaisse M, Friedlander G, Houillier P, Maruani G (2008). Update on vitamin D and evaluation of vitamin D status. Ann Endocrinol.

[7] Adams JS, Hewison M (2010). Update in vitamin D. J Clin End Metab.

[8] Mulligan ML, Felton SK, Riek AE, Bernal-Mizrachi C (2010). Implications of vitamin D deficiency in pregnancy and lactation. Am J Obs Gyn.

[9] Shibata M, Suzuki A, Sekiya T, Sekiguchi S, Asano S, Udagawa Y (2011). High prevalence of hypovitaminosis D in pregnant Japanese women with threatened premature delivery. J Bone Miner Metab.

[10] Tao M, Shao H, Gu J, Zhen Z (2012). Vitamin D status of pregnant women in Shanghai, China. J Maternal-Fetal and Neonatal Medicine.

[11] Perampalam S, Ganda K, Chow KA, Opie N, Hickman PE, Shadbolt B (2011). Vitamin D status and its predictive factors in pregnancy in 2 Australian Populations. Australian and New Zealand. J Obs and Gyn.

[12] Winters SJ, Chennubhatla R, Wang C (2009). Influence of obesity on vitamin D-binding protein and 25-hydroxy vitamin D levels in African American and white women. Metabolism clinical and experimental.

[13] Pehlivan I, Hartun S, Aydogan M (2003). Maternal vitamin D deficiency and vitamin D supplementation in healthy infants. Turk J Pediatr.

[14] Lips P (2007). Vitamin D status and nutrition in Europe and Asia. J steroid Bioch Molec Biol.

[15] Belaid S, Martin A, Schott AM (2008). Hypovitaminosis D among 18 to 49 years old women wearing concealing clothes, an ignored reality in general practice. Press Med.

[16] Mbou FM, Pages-Lutz F, Garabedian M, Walrant-Debray O, Leguyader P, Robert P (2009). Status vitaminique D maternal in Martinique. J Gyn Obst Biol Reprod.

[17] Bodnar LM, Simhan HN, Powers RW (2007). High Prevalence of Vitamin D Insufficiency in Black and white pregnant women residing in the Northern United States and Their Neonates. Americ Society for Nutrition.

[18] Kalra P, Das V, Agarwal A, Kumar M, Ramesh V, Bhatia E (2012). Effect of vitamin D supplementation during pregnancy on neonatal mineral homeostasis and anthropometry of the newborn and infant. Br J Nut.

